# Association between sarcopenia and low back pain in local residents prospective cohort study from the GAINA study

**DOI:** 10.1186/s12891-017-1807-7

**Published:** 2017-11-15

**Authors:** Shinji Tanishima, Hiroshi Hagino, Hiromi Matsumoto, Chika Tanimura, Hideki Nagashima

**Affiliations:** 1 Department of Orthopedic Surgery,Faculty of Medicine, Tottori University, 36-1 Nishi-cho, Yonago, Tottori, 683-8504 Japan; 2 School of Health Science, Tottori University Faculty of Medicine, 86 Nishi-cho, Yonago, Tottori, 683-8503 Japan; 3 Rehabilitation Division, Tottori University Hospital, 36-1 Nishi-cho, Yonago, Tottori, 683-8504 Japan

**Keywords:** Sarcopenia, Low back pain, Muscle strength, Osteoporosis

## Abstract

**Background:**

Low back pain (LBP) is one of the most common ailments that people experience in their lifetime. On the other hands, Sarcopenia also leads to several physical symptoms and contributes to reducing the quality of life of elderly people.The purpose of this study is to investigate the association between sarcopenia and low back pain among the general population.

**Methods:**

The subjects included 216 adults (79 men and 137 women; mean age, 73.5 years) undergoing a general medical examination in Hino, Japan. Skeletal muscle index (SMI), The percentage of young adults’ mean (%YAM) of the calcaneal bone mass using with quantitative ultrasound (QUS) method and walking speed were measured, and subjects who met the criteria of the Asian Working Group for Sarcopenia were assigned to the sarcopenia group. Subjects with decreased muscle mass only were assigned to the pre-sarcopenia group, and all other subjects were assigned to the normal group. Then, we compared the correlations with low back pain physical finding. The Oswestry Disability Index (ODI) and the low back pain visual analogue scale (VAS) were used as indices of low back pain. Statistical analysis was performed among three groups with respect their characteristic, demographics, data of sarcopenia determining factor, VAS and ODI. We also analysed prevalence of LBP and sarcopenia. We investigated the correlations between ODI and the sarcopenia-determining factors of walking speed, muscle mass and grip strength.

**Results:**

Sarcopenia was noted in 12 subjects (5.5%). The pre-sarcopenia group included 38 subjects (17.6%), and the normal group included 166 subjects (76.9%). The mean ODI score was significantly higher in the sarcopenia group (25.2% ± 12.3%; *P* < 0.05) than in the pre-sarcopenia group (11.2% ± 10.0%) and the normal group (11.9% ± 12.3%). %YAM and BMI were significantly lower in the sarcopenia group than in other groups (*P* < 0.05). A negative correlation existed between walking speed and ODI (*r* = −0.32, *P* < 0.001).

**Conclusions:**

The results of this study suggested that decreased physical ability due to quality of life in residents with LBP may be related to sarcopenia.

## Background

Low back pain (LBP) is one of the most common ailments that people experience in their lifetime. The lifetime prevalence of LBP is approximately 84% [1]. LBP is caused by many factors. Wan et al. reported that muscle atrophy may lead to chronic LBP at multiple levels of the lumbar spine [2]. Several studies have reported that atrophy of the back muscles is a factor that causes LBP [2–4]; however, no consensus has been reached on an association between sarcopenia and LBP. Sarcopenia is defined as the pathophysiology caused by decreased muscle strength accompanying ageing [5]. Sarcopenia results in several disorders, such as hypertension, obesity, osteoporosis and diabetes mellitus [6, 7]. Sarcopenia also leads to several physical symptoms and contributes to reducing the quality of life of elderly people [8, 9].

The relationship between sarcopenia and LBP is unclear, and has been explored by only few studies. The current study aimed to clarify the association between sarcopenia and LBP.

## Methods

### Subjects

This study was based on the results obtained from a prospective cohort of subjects enrolled in the Good Ageing and Intervention Against Nursing Care and Activity Decline (GAINA) study. The GAINA study, which began in 2014, is a population-based study of cohorts from Hino, Tottori Prefecture, Japan. The population comprised 3352 subjects in September 2016, with an ageing rate of approximately 45%. The subjects were recruited from individuals who underwent an annual town-sponsored medical check-up. A self-administered questionnaire was sent to 1450 subjects aged >40 years who were eligible to receive the medical check-up. We sent the consent form for The GAINA study together with medical check-up form to all subjects before an annual town-sponsored medical check-up. We enrolled the subjects who agreed The GAINA study. The baseline assessment was performed between May and June 2014 on 273 individuals undergoing the medical check-up. The inclusion criteria for subjects in the study were 1) living independently, 2) the ability to walk to where the survey was performed and 3) agreement to provide self-reported data. Fifty-seven subjects were excluded for lack of data because of omission of recording of medical check-up form. A total of 216 subjects (79 men and 137 women) participated in the baseline assessment. All subjects provided written informed consent, and the study was approved by the local ethics committee of the Faculty of Medicine, Tottori University (No. 2354).

### Baseline measurements

Baseline characteristics, such as age, sex, height, body weight, body mass index (BMI), smoking habit and alcohol habit were recorded. We regarded the subjects who answered “yes” against this question “Do you feel low back pain in your daily life lately?” were LBP subjects. The position of low back was defined by each subject. Subjects were asked to make a vertical mark through a 100-mm horizontal VAS Scale.

We also used the Oswestry Disability Index (ODI) to assess functional outcomes associated with LBP. The results of the self-administered questionnaire were then checked for accuracy by researchers who personally interviewed each subject.

### Assessment of sarcopenia

The participants were classified as having sarcopenia based on muscle mass, muscle strength and physical performance. The classification was based on the recommendations of the Asian Working Group for Sarcopenia [10]. The Recommendations of the Asian Working Group for Sarcopenia defined Sarcopenia as the Subjects were classified as having sarcopenia if they were aged >60 years and had a low handgrip strength (<26 kg in men and <18 kg in women) and/or a lower walking speed (<0.8 m/s) with a low muscle mass (<7.0 kg/m^2^ in men and 5.7 kg/m^2^ in women).

**Fig. 1:**
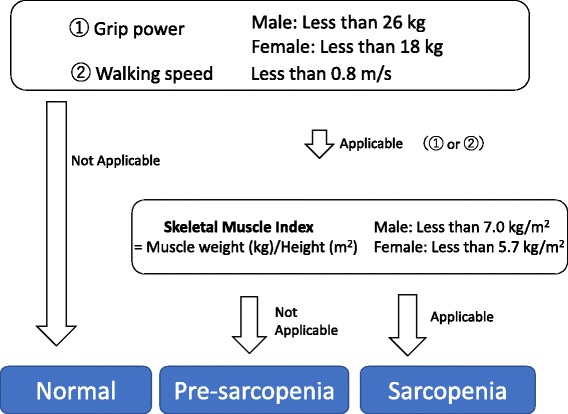
Classification of study subjects. The subjects were divided into three groups by the Asian Working Group for Sarcopenia. Subjects with decreased muscle mass only were assigned to the pre-sarcopenia group and all other subjects were assigned to the normal group

In this study, some subjects had low muscle mass under 60 years, so we defined subjects were classified as having sarcopenia if they were aged >40 years and had a low handgrip strength (<26 kg in men and <18 kg in women) and/or a lower gait speed (<0.8 m/s) with a low muscle mass (<7.0 kg/m^2^ in men and 5.7 kg/m^2^ in women). Subjects were classified as having pre-sarcopenia if they were aged >40 years and had a low handgrip strength (<26 kg in men and <18 kg in women) and/or a lower walking speed (<0.8 m/s) without a low muscle mass (<7.0 kg/m^2^ in men and 5.7 kg/m^2^ in women). Subjects without low muscle mass or strength or low physical performance were classified as normal (Fig. 1).

### Body function and structure measurements

Handgrip strength was measured using a TKK 5401 dynamometer (Takei Co, Niigata City, Japan). The subjects were asked to squeeze the dynamometer twice with each hand. The highest scores for the left and right hands were summed. Muscle mass was measured by bioelectrical impedance analysis (BIA) with a MC-780A Body Composition Analyzer (Tanita Co., Tokyo, Japan). The BIA method requires the subjects onto a platform and remain in the standing position for approximately 30 s. Skeletal mass index was calculated by dividing the limb muscle mass (kg) by the square of the height (m). We used quantitative ultrasound (QUS) to assess the calcaneal bone mass [11, 12]. The speed of sound through the calcaneus was evaluated using a CM-200 sonometer (Furuno Co., Nishinomiya City, Japan). The subject was seated and was asked to place the right heel on the QUS device. Coupling gel was applied to the heel to facilitate the transmission of ultrasound to the skeletal site being examined. The sum of the percentage of young adult mean was calculated. Gait parameters were obtained using the Opto Gait (Microgate Co., Bolzano, Italy) designed for optical-sensitive gait analysis. We prepared a 10-m walking line. Walking section and measurement section were set respectively. The subjects completed a single trial at free speed with the instruction to ‘walk at your normal speed ‘. Walking speed was calculated with specific software (OPTO Gait analysis software, version 1.6.4.0, Microgate S.r.L, Italy).

### Statistical analysis

All data were expressed as mean ± standard deviation. The subjects were divided into normal, pre-sarcopenia and sarcopenia groups. Differences characteristic, demographics, data of sarcopenia determining factor, VAS and ODI of the subjects among three groups were examined using Steel-Dwass test. The differences of prevalence of LBP among three groups analysed with Chi-square for independence test, m × n contingency table.

The differences of prevalence of LBP between men and women analysed with the Pearson’s chi-square test or Fisher’s exact test. We performed Fisher’s exact test when expected cell size is <5**.** To investigate the correlations between ODI and the sarcopenia-determining factors, we used a partial correlation analysis with controlling the age and BMI available.

Data were analysed with StatMate for windows, version 4.01 (ATMS Corporation, Tokyo, Japan).

## Results

### Prevalence of sarcopenia

The prevalence of sarcopenia was approximately 5.5% (12 subjects; 5 men and 7 women). %YAM and BMI were significantly lower in the sarcopenia group than in the Normal groups. BMI in the Pre-sarcopenia group were significantly lower in the other groups (Table 1). The prevalence of sarcopenia in men and women were 8.4% in men and 4.3% in women. There was not significantly different in gender (Chi-square test; *P* = 0.32, data not shown).

**Table 1:** Characteristic and demographics of the subjects

	Normal	Pre-sarcopenia	Sarcopenia
	(*n* = 166)	(*n* = 38)	(*n* = 12)
Age (years)	73.0 ± 7.8	72.2 ± 8.5	84.9 ± 5.0 **
Gender (M:F)	63:103	11:27	5:7
%YAM (%)	78.9 ± 13.7	78.3 ± 15.9	63.8 ± 8.7 **
BMI (Kg/m^2^)	22.8 ± 2.3	18.9 ± 2.0**	20.6 ± 2.4*
Smoking habit (%)	25.6	26.3	8.3
Alcohol habit (%)	30.1	42.1	25.0

(Mean ± SD)

Steel-Dwass *P < 0.05,**P < 0.01

%YAM: the percentage of young adult mean

BMI: Body mass index

M:male

F: female

Sarcopenia was noted in 12 of 216 subjects (5.5%). %YAM and BMI were significantly lower in the sarcopenia group than in the Normal groups. BMI in the Pre-sarcopenia group were significantly lower in the other groups. %YAM and BMI were significantly lower in the sarcopenia group than in the other groups (Steel-Dwass test, *: *P* < 0.05, **:*P* < 0.01). There were no significant differences among the three groups about smoking habit and alcohol habit (Chi-square for independence test, m × n contingency table)

### Prevalence of LBP

One hundred-forty out of 216 subjects complained LBP. The overall prevalence of complaints of LBP was 64.8% (140/216 subjects). More than 60% of the subjects in each group complained of LBP. The prevalence of LBP was not significantly different among the three groups (Table 2). The prevalence LBP in men and women were 72.2% (52/79 subjects) and 65.0% (88/137 subjects). There was not significantly different in gender (Chi-square test; *P* = 0.89, data not shown).

**Table 2:** Prevalence of low back pain

	LBP(−)	LBP(+)	Prevalence of LBP (%)
Normal (*n* = 166)	61	105	60.1 (105/166)
Pre-sarcopenia (*n* = 38)	13	25	65.8 (25/38)
Sarcopenia (*n* = 12)	2	10	83.3 (10/12)
Total (*n* = 216)	76	140	64.8 (140/216)

There were no significant differences among the three groups

### Sarcopenia and LBP

The mean VAS score was the highest in the sarcopenia group, although there were no significant differences among the groups. The mean ODI score in sarcopenia group was 24.3%. This score was significantly higher in the Sarcopenia group than in the other groups. The mean walking speed in the sarcopenia group was significantly lower than in the other groups. Grip power in the Pre-sarcopenia and Sarcopenia group were significantly lower than in the normal group. SMI in the Pre-sarcopenia and Sarcopenia group were significantly lower than in the normal group (6.9kg/m^2^ in Pre-sarcopenia and 6.5kg/m^2^ in Sarcopenia vs 6.9kg/m^2^ in Normal, *P* < 0.05) (Table 3).

**Table 3:** Sarcopenia and low back pain

	Normal (*n* = 166)	Pre-sarcopenia (*n* = 38)	Sarcopenia (*n* = 12)
VAS (mm)	20.5 ± 25.4	21.3 ± 25.8	23.5 ± 22.0
ODI (%)	11.9 ± 12.3	11.2 ± 10.0	25.2 ± 12.3 **
Walking speed (m/s)	1.2 ± 0.3	1.3 ± 0.3	0.9 ± 0.4**
Grip power (kg)	29.8 ± 8.3	26.3 ± 6.4*	20.7 ± 6.0**
SMI (Kg/m^2^)	7.0 ± 0.9	5.8±0.7**	6.1±0.6**

(Mean ± SD)

Steel-Dwass

*P < 0.05 **P < 0.01

Oswestry Disability Index scores were significantly higher in the sarcopenia group than in the other groups (*P* < 0.05). The mean visual analogue scale score in the sarcopenia group was the highest among the three groups, although there were no significant differences among the groups. The mean walking speed in the sarcopenia group was significantly lower than in the other groups. Grip power in the Pre-sarcopenia and Sarcopenia group were significantly lower than in the normal group. SMI in the Pre-sarcopenia and Sarcopenia group were significantly lower than in the normal group

### Association between sarcopenia and ODI

We investigated the correlations between ODI and the sarcopenia-determining factors of walking speed, muscle mass and grip strength. The only correlation was a negative correlation with walking speed (correlation confident −0.32, *P* < 0.001) (Table 4).

**Table 4:** Association between sarcopenia and Oswestry Disability Index (ODI)

	Correlation coefficient	*P* Value
Walking speed	−0.32	<0.001
Grip power	−0.26	0.05
Skeletal muscle index	−0.26	0.70

(Partial correlation analysis: control the age and BMI variable)

BMI:Body mass index

The only relationship was a negative correlation between walking speed and ODI. (Partial correlation analysis: control the age and BMI variable)

## Discussion

We investigated the association between sarcopenia and LBP in local residents, focussing on elderly people. Sarcopenia was defined as ‘age-related loss of muscle mass and function’ by Rosenberg [5]. Musculoskeletal disorders are greatly influenced by sarcopenia. Baumgartner et al. reported that the prevalence of sarcopenia was more than 50% in people aged >80 years in Mexico and that more people with sarcopenia had physical disabilities [13]. Janssen et al. reported that fifth decades people begin to start decreasing their muscle volume [14] and people with low skeletal muscle mass index existed in third to sixth decades with same prevalence of over six decades in their study for 4504 American adults [15]. We included residents who are around fifth decades in this study for this reason.

The prevalence of sarcopenia was only 12% in this study, this was lower than other study. The inclusion criteria for subjects in the study were 1) living independently, 2) the ability to walk to where the survey was performed.

This criterion might affect that low prevalence of sarcopenia in this study.

LBP is one of the most common symptoms treated in daily medical practice. Park et al. investigated the prevalence of sarcopenia and lumbar spinal stenosis in Korea [16]. The prevalence of sarcopenia was higher in people with lumbar spinal stenosis than in normal people. They suggested that LBP with lumbar spinal stenosis led to low physical activity, causing sarcopenia. Although the present study showed that the prevalence of LBP was not significantly different among the three groups, the ODI scores were significantly higher in the sarcopenia group than in the other groups. The mean VAS score in the sarcopenia group was the highest among the three groups, although there were no significant differences among the groups.

The overall prevalence rate of LBP was 64.8%. Suka et al. performed a big survey for 3048 men and 1885 women in Japan to investigate the prevalence rate of LBP. They reported the prevalence rate of LBP was 26.5%. This prevalence was lower than our study [17]. In this study, most subjects over seventies and work as a former in this study, this situation might have relationship with high prevalence rate of LBP.

We consider that sarcopenia is not the cause of LBP. However, we focused on LBP in this study. LBP is induced by many factors, such as osteoporosis and muscle disorders [18]. The sarcopenia group in this study had low %YAM and BMI. The average %YAM in the sarcopenia group was 63.8% ± 8.7%. Verschueren et al. reported that sarcopenia was associated with low BMD in middle-aged and elderly European men [19]. In Japan, under 70% YAM is one of the criteria for osteoporosis [20]. Based on this criterion, most subjects with sarcopenia in our study may have had osteoporosis. Generally, the prevalence of osteoporosis is higher in elderly women than in men [21, 22]. The fact that more than 50% of the subjects in this study were women may affect these results. It is a well-known fact that osteoclasts are highly active in osteoporosis. The relationship between bone cancer pain and osteoclast activity is well known [23, 24]. There are no reports showing a relationship between osteoclast activity and osteoporotic bone pain.

In the periosteum, the A-delta and C-sensory nerve fibres are arranged in a fishnet-like pattern, which appears to be designed to act as a “neural net” to detect mechanical injury or distortion of the underlying cortical bone [25]. Park et al. mentioned that the following mechanisms contribute to generating and maintaining pain in osteoporosis: 1) the increasing density of the bone sensory nerve fibres in the elderly; 2) the expression of nociceptors by sensory nerve fibres sensitised by lower pH (as observed during osteoclastic activity) and 3) pathological modifications of bone sensory nerve fibres. The periosteum receives more sensory innervation than any other part of the skeleton [26]. We did not investigate the mechanism of LBP induced by osteoporosis in this study. As most subjects were elderly women, the presence of osteoporosis could not be neglected. We consider that the subjects who complained of LBP had low bone mineral density (BMD)-induced bone pain, especially elderly women.

We also investigated correlations between ODI and the sarcopenia-determining factors of walking speed, muscle mass and grip strength. ODI was associated with walking speed. Muscle power and volume did not affect LBP. Previous studies reported that exercising the lumbar muscles improved chronic LBP [27, 28].

We did not assess the volume and strength of the lumbar muscles and the effect of lumbar exercise of local residents. The relationship between the strength of these muscles and LBP is unclear. Among the sarcopenia-determining factors, only walking speed correlated with ODI.

Low walking speed associated ODI. We consider that low walking speed equates to low physical ability. Low physical activity might have association with low muscle volume and %YAM. We consider that low physical ability may associate with sarcopenia. In this study, although we could not be determined whether low physical activity is a cause or a result of sarcopenia and osteoporosis, measures against low physical ability, such as exercise and osteoporosis therapy, may help prevent sarcopenia.

This study had several limitations. First, being a cross-sectional study, it did not reveal the causal relationships between sarcopenia and LBP. We did not investigate the causes of LBP without sarcopenia, such as disc herniation, lumbar spinal stenosis and spinal deformity. Second, the study may have had a subject selection bias because the subjects voluntarily participated in the medical check-up. We performed this study in a small town in mountains and most of subject who were recruited were over 70’s. Our inclusion criteria were living independently and the ability to walk to where the survey was performed. As a result, subjects who could attend the check-up may have had higher levels of activities of daily living. These factors affect the result of our research as a selection bias. Especially, this bias may have relationship with the low prevalence of sarcopenia in this study. Third, sample size was too small.

## Conclusion

Low back pain was associated with osteoporosis and cause low physical activity. As a result, these situations caused sarcopenia. We consider that exercise against low physical activity and osteoporosis therapy may affect sarcopenia.

## Abbreviations

%YAM: The percentage of young adult mean
BIA: Bioelectrical impedance analysis
BMD: Bone mineral density
GAINA: Good Ageing and Intervention Against Nursing Care and Activity Decline
LBP: Low back pain
ODI: Oswestry Disability Index
QUS: Quantitative ultrasound
SMI: Skeletal muscle index
VAS: Visual analogue scale
